# Less‐Invasive Capsulotomy Strategy in Hip Arthroscopy: The Gluteus Minimus Sign as a Visual Landmark for the Joint Capsule Based on Cadaveric Analysis

**DOI:** 10.1002/atn2.70139

**Published:** 2026-05-23

**Authors:** Tetsuya Tachibana, Masahiro Tsutsumi, Akimoto Nimura, Hajime Utsunomiya

**Affiliations:** ^1^ Department of Orthopedic Surgery, Dokkyo Medical University Saitama Medical Center Saitama Japan; ^2^ Inclusive Medical Sciences Research Institute Morinomiya University of Medical Sciences Osaka Japan; ^3^ Department of Clinical Anatomy Institute of Science Tokyo Tokyo Japan; ^4^ Department of Functional Joint Anatomy Institute of Science Tokyo Tokyo Japan; ^5^ Tokyo Research Institute for Sports Medicine Chiba Japan

## Abstract

Hip arthroscopy requires a capsulotomy to achieve adequate visualization and access; however, an excessive incision can increase the risk of postoperative instability. This technical note describes a less‐invasive capsulotomy technique guided by the “gluteus minimus sign,” a fibrous membrane connecting the main tendon of the gluteus minimus to the joint capsule. Cadaveric analysis confirmed that this fibrous membrane corresponds to the anterosuperior region of the femoral neck. Most gluteus minimus functions can be preserved by limiting the anterior extension of the capsulotomy to within this sign. When additional anterior exposure is necessary, superficial tendon fibers can be incised using an arthroscopic knife. This technique offers a safe, anatomically informed, and strategically targeted capsulotomy that balances preservation of joint stability with adequate surgical access.

## Graphical abstract

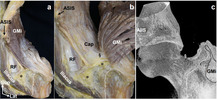

**VIDEO 1:** Arthroscopic demonstration of a less‐invasive capsulotomy technique based on the gluteus minimus sign in a left hip. The patient is in the supine position, and the procedure is performed using a 70° arthroscope. The video shows portal placement, creation of a skip capsulotomy (a transverse incision from the anterolateral portal and a separate small incision at the mid‐anterior portal), identification of the gluteus minimus sign as an extra‐articular landmark indicating the anterosuperior portion of the joint capsule, and controlled anterior extension of the capsulotomy based on this landmark. Subsequent steps include labral repair, cam resection, and capsular plication. This technique illustrates a balanced capsulotomy strategy that preserves capsular integrity while maintaining adequate visualization by considering both the size and location of the capsular incision. Video content can be viewed at https://doi.org/10.1002/atn2.70139.

Hip arthroscopy has become the gold standard treatment for femoroacetabular impingement and labral tears. Owing to the anatomical constraints of the hip joint, adequate visualization requires joint distraction and capsulotomy.^1^ The most commonly employed technique is interportal capsulotomy, which involves a transverse incision between the anterolateral portal (ALP) and mid‐anterior portal (MAP).^2^ However, this approach is associated with the risk of postoperative hip instability.^3^, ^4^, ^5^ To prevent iatrogenic instability, alternative techniques, such as T‐capsulotomy and periportal capsulotomy, have been proposed to preserve the iliofemoral ligament.^6^, ^7^ Although the extent of capsulotomy is an important determinant of postoperative stability, an overly limited incision may hinder visualization and access, thereby compromising complete resection of cam deformities.^8^ Therefore, achieving a balance between sufficient exposure and the preservation of stability remains a major challenge in hip arthroscopy.

Recent anatomical studies of the hip joint capsule have revealed that the iliofemoral ligament is not an isolated cord‐like structure such as the anterior cruciate ligament, but rather a thickened portion of the joint capsule itself formed through fibrous continuity with the gluteus minimus tendon and the deep aponeurosis of the iliopsoas muscle.^9^ These findings indicate that not only the size but also the location of the capsulotomy is critical for optimal capsular management. Because the intra‐articular appearance of the capsule provides few reliable landmarks, surgeons often face difficulties in determining the precise incision site. According to an anatomical study, the deep aponeurosis of the gluteus minimus partly forms a fibrous membrane connecting the main tendon of the gluteus minimus and the joint capsule in the anterosuperior region of the femoral neck^9^ (Figure 1a‐c). Based on these anatomical findings, we identified this fibrous membrane as a useful extra‐articular landmark for determining the capsulotomy location. In this technical note, we describe the arthroscopic appearance of this fibrous membrane, known as the “gluteus minimus sign.” This sign can serve as a useful landmark to guide strategically limited, less‐invasive capsulotomies.

**FIGURE 1:**
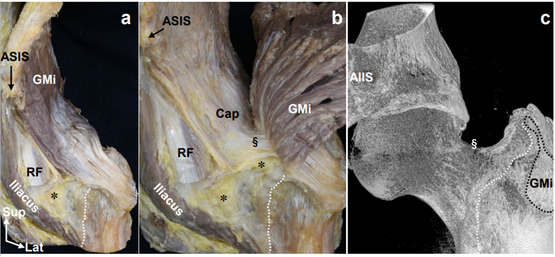
Cadaveric dissection of the anterior capsule of a left hip. (a) Anterior view of the left hip after removal of the superficial portions of the gluteus minimus (GMi), rectus femoris (RF), and iliopsoas muscles. The distal portion of the RF was additionally removed to expose the fat pad and loose connective tissue overlying the anterior joint capsule (asterisk). The white dotted line indicates the intertrochanteric line. (b) After detachment of the origin of the gluteus minimus, the deep aponeurosis of the gluteus minimus is identified as a fibrous membrane (section sign) connecting the gluteus minimus tendon to the joint capsule (Cap). This fibrous structure is defined as the “gluteus minimus sign.” (c) Corresponding microcomputed tomography images of the same specimen. The black dotted line indicates the insertion of the gluteus minimus tendon. Correlation between the cadaveric dissection and micro‐CT images shows that the gluteus minimus sign is located on the anterosuperior portion of the joint capsule. Limiting anterior capsulotomy to within this landmark may help preserve joint stability and gluteus minimus function. (AIIS, anterior inferior iliac spine, ASIS, anterior superior iliac spine, Ant, anterior, Lat, lateral.). (This figure was newly created based on the anatomical findings previously reported in Tsutsumi et al. [2020].^9^)

## SURGICAL TECHNIQUE

The procedure is performed with the patient in the supine position on a postless traction table,^10^ allowing for dynamic leg manipulation.^11^ Both lower limbs are padded, secured in traction boots, and gently distracted by a distance of 1 to 2 cm.

**FIGURE 2:**
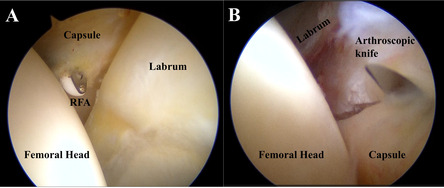
Skip capsulotomy in a left hip. The patient is in the supine position, and arthroscopic images are obtained using a 70° arthroscope. (A) Arthroscopic view from the ALP showing creation of a 5 mm capsulotomy at the MAP using a RFA device. (B) Arthroscopic view from the MAP showing creation of a separate 15 to 20 mm capsulotomy at the ALP using an arthroscopic knife, resulting in a noncontinuous “skip” capsulotomy configuration. (ALP, anterolateral portal; MAP, mid‐anterior portal; RFA, radiofrequency ablation.)

The ALP is first established under fluoroscopy, and a 70° arthroscope is introduced into the central compartment. The MAP is created under direct visualization. A 5 mm longitudinal capsulotomy is performed in the MAP using a radiofrequency ablation (RFA) device (Smith & Nephew, Andover, MA, USA) (Figure 2A). A separate transverse capsulotomy from the ALP is extended 5 to 10 mm anteriorly toward the MAP and 10 mm posteriorly using an arthroscopic knife (Beaver‐Visitec International, Waltham, MA, USA) (Figure 2B). We have termed these 2 noncontinuous capsulotomies as the “skip capsulotomy.”^10^

After completing the central compartment procedures—including separation of the capsule above the labrum, subspine decompression with a 4.5‐mm curved burr (ConMed, Largo, FL, USA), and labral repair using 1.8‐mm Q‐FIX curved suture anchors (Smith & Nephew, Andover, MA, USA)—the arthroscope is redirected into the joint capsule via the ALP.

**FIGURE 3:**
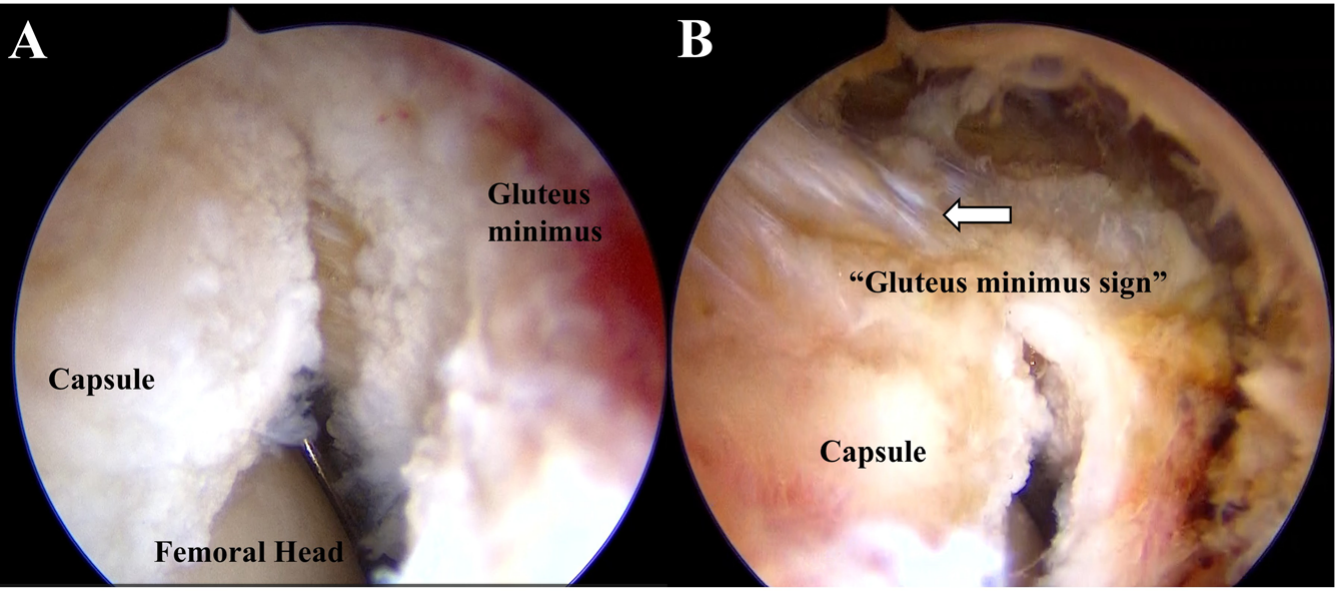
The gluteus minimus sign in a left hip. The patient is in the supine position, and arthroscopic images are obtained using a 70° arthroscope. (A) Arthroscopic view from the ALP showing the cut ends of the medial side of the skip capsulotomy on the outer surface of the joint capsule. The gluteus minimus muscle and loose connective tissue overlying the capsule have been removed using a RFA device. (B) Arthroscopic view after removal of loose connective tissue at the capsular incision site, revealing a distinct fibrous membrane (arrow) connecting the gluteus minimus tendon to the joint capsule. This fibrous structure represents the “gluteus minimus sign” and serves as an extra‐articular landmark for guiding anterior capsulotomy. Limiting anterior capsulotomy to within this landmark may help prevent iatrogenic instability while preserving most of the function of the gluteus minimus muscle. (ALP, anterolateral portal; RFA, radiofrequency ablation.)

Under ALP visualization, the RFA is introduced through the MAP. Both the RFA and scope are gradually withdrawn from the intra‐articular space to the extra‐articular surface of the capsule. The cut ends of the proximal and distal capsules are first observed (Figure 3A). As the scope is withdrawn slightly further, it reaches the extra‐articular space where the gluteus minimus muscle attaches to the capsule surface. Removal of the gluteus minimus muscle using the RFA exposes the fibrous membrane connecting the gluteus minimus tendon and the joint capsule (Figure 3B). These structures form parallel bundles of thick fibers, which we termed “the gluteus minimus sign” (Video 1).

**FIGURE 4:**
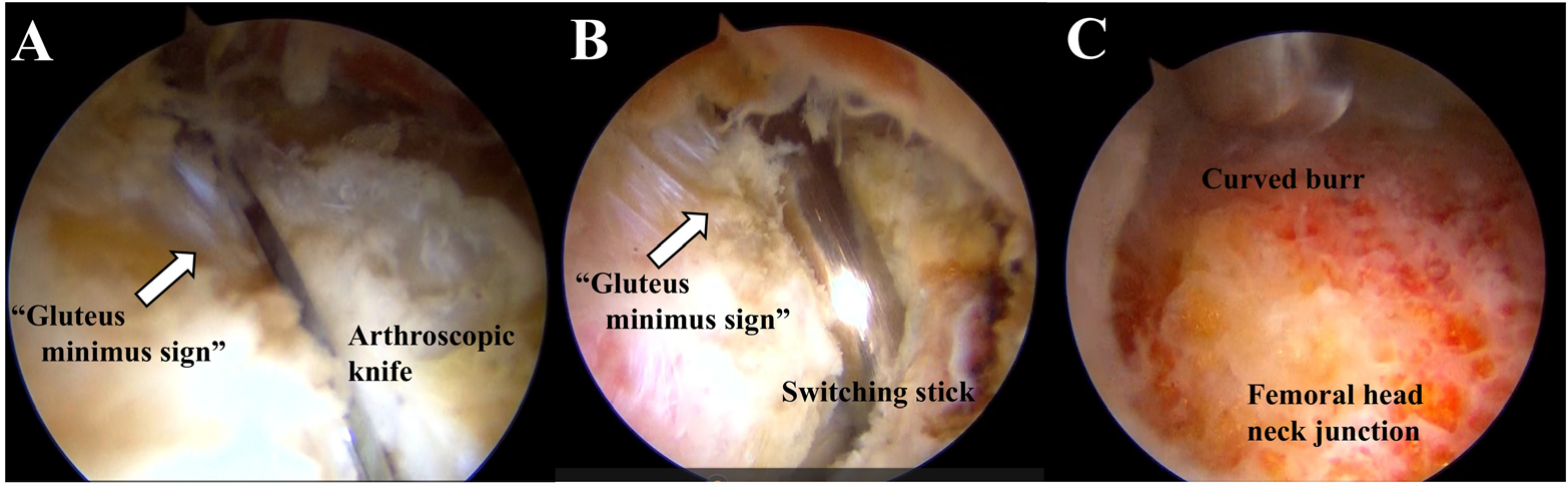
Technique for extending the capsulotomy under visual control using the gluteus minimus sign in a left hip. The patient is in the supine position, and arthroscopic images are obtained using a 70° arthroscope. (A) Arthroscopic view from the ALP showing incision of the superficial fibers of the gluteus minimus sign using an arthroscopic knife, allowing controlled anterior extension of the capsulotomy while preserving deeper capsular structures. (B) Arthroscopic view from the ALP showing anterior extension of the skip capsulotomy under direct visualization of the gluteus minimus sign, with insertion of a switching stick through the mid‐anterior portal to confirm adequate joint access. (C) Arthroscopic view from the ALP showing excellent visualization and instrument maneuverability during cam resection using a curved burr at the femoral head‐neck junction through the skip capsulotomy. (ALP, anterolateral portal.)

The gluteus minimus sign is located on the anterosuperior portion of the capsule and provides the surgeon with precise information regarding the capsulotomy location. By limiting the anterior extension of the capsulotomy to within this range, most of the function of the gluteus minimus can be preserved. However, in certain cases, particularly when resection of a pronounced cam morphology is required, additional anterior extension of the capsulotomy may be necessary. If the insertion of a switching stick through the MAP into the intra‐articular space is difficult, the capsulotomy can be extended anteriorly by incising the superficial fibers of the gluteus minimus tendon using an arthroscopic knife (Figure 4A,B). Surgeons should carefully control the anterior extension of the capsulotomy while keeping in mind that a complete full‐thickness incision is generally unnecessary. These maneuvers allow the surgeon to control not only the size, but also the location of the capsulotomy based on the gluteus minimus sign. Both the curved burr and the arthroscope are inserted and manipulated within the same capsulotomy to correct the cam deformity (Figure 4C). Finally, capsular closure is performed at the anterior portion of the capsulotomy, where the gluteus minimus tendon is attached, using 1 or 2 sutures, employing either the Quebec City slider or shoelace technique.^11^, ^12^

## DISCUSSION

In this technical note, we describe a capsulotomy technique that enables strategic extension of the incision based on the concept that both the size and location of the capsulotomy are critical for proper capsular management. Conventional techniques, including periportal and T‐shaped capsulotomies, have primarily focused on the size of the capsular incision. However, little attention has been paid to its location. The iliofemoral ligament is a thickened portion of the joint capsule that is fibrously continuous with the gluteus minimus tendon and the deep aponeurosis of the iliopsoas.^9^ These findings suggest that observing musculotendinous attachments to the capsule can provide important information regarding capsulotomy location. The gluteus minimus sign, a fibrous membrane partly formed by the deep aponeurosis of the gluteus minimus, is located in the anterosuperior portion of the capsule. The gluteus minimus sign is considered to play an important functional role in joint stability by transmitting tension from the gluteus minimus to the joint capsule, thereby pulling the femoral head toward the acetabulum.^13^, ^14^, ^15^ Therefore, our strategy of performing a limited additional anterior capsular incision after identifying the gluteus minimus sign in the extra‐articular space can provide a functionally less‐invasive and well‐balanced capsulotomy that offers adequate arthroscopic visualization.

The anterior incision should be limited to within the gluteus minimus sign to minimize postoperative instability but can be extended to this landmark if additional exposure is required. When extending the capsulotomy, RFA may result in partial loss of the capsule. To avoid this, it is preferable to use an arthroscopic knife and gently shave the superficial fibers of the gluteus minimus tendon (Table 1). The advantages of this technique include preservation of capsular integrity and easier capsular closure due to exposure of the outer surface of the capsule. Despite these advantages, several limitations of this technique should be acknowledged. Identification of the gluteus minimus sign requires extra‐articular exposure of the capsule and may be technically demanding, particularly in cases with severe adhesions. In addition, limiting the anterior extension of the capsulotomy may restrict visualization in patients with pronounced cam morphology, occasionally necessitating careful incision of the superficial fibers of the gluteus minimus tendon (Table 2).

**TABLE 1:** Pearls and Pitfalls

**Pearls**	**Pitfalls**
Use the gluteus minimus sign as a reliable extra‐articular landmark to control capsulotomy	Excessive anterior extension beyond the gluteus minimus sign increases the risk of postoperative instability
Limit the anterior incision to within this sign to preserve most of the function of the gluteus minimus	Overuse of RFA during capsulotomy extension may result in partial capsule loss
When additional exposure is required, incise only the superficial fibers of the gluteus minimus tendon with an arthroscopic knife	Insufficient capsulotomy may limit visualization and hinder complete resection of cam morphology
Posterior extension of the capsulotomy from the ALP is generally safe due to the absence of muscular attachments in this area	

ALP, anterolateral portal; RFA, radiofrequency ablation.

**TABLE 2:** Advantages and Disadvantages

**Advantages**	**Disadvantages**
Preservation of capsular integrity, reducing the risk of postoperative instability	Requires extra‐articular approach to identify the gluteus minimus sign
Ability to selectively extend the capsulotomy anteriorly when additional exposure is required	Limited anterior extension may restrict visualization in cases of pronounced cam morphology
Easier capsular closure due to exposure on the outer surface of the capsule	Identification of the landmark may be challenging in patients with severe adhesions

Moreover, anatomical studies have shown that the posterior portion of the ALP in the joint capsule is generally thin and lacks muscular attachment until the obturator internus tendon's attachment.^9^ This anatomical characteristic suggests minimal functional compromise from posterior extension of the capsulotomy. Therefore, we believe that extending the capsulotomy posteriorly during cam resection remains an acceptable and functionally safe option.

## DISCLOSURES

The authors (T.T., M.T., A.N., H.U.) declare that they have no known competing financial interests or personal relationships that could have appeared to influence the work reported in this paper.
